# A Machine Learning Method to Trace Cancer Primary Lesion Using Microarray-Based Gene Expression Data

**DOI:** 10.3389/fonc.2022.832567

**Published:** 2022-04-21

**Authors:** Qingfeng Lu, Fengxia Chen, Qianyue Li, Lihong Chen, Ling Tong, Geng Tian, Xiaohong Zhou

**Affiliations:** ^1^ Oncology Department, Daqing Oilfield General Hospital, Daqing, China; ^2^ Department of Thoracic Surgery, Hainan General Hospital, Haikou, China; ^3^ Department of R&D, Geneis (Beijing) Co., Ltd., Beijing, China; ^4^ Department of Emergency, Qingdao Eighth People’s Hospital, Qingdao, China; ^5^ Department of Pathology, Chifeng Municipal Hospital, Chifeng Clinical Medical School of Inner Mongolia Medical University, Chifeng, China; ^6^ Second Division of Cancer, Jiamusi Cancer Hospital, Jiamusi, China

**Keywords:** cancer of the unknown primary site, human malignancies, gene expression, XGBoost, gene selection

## Abstract

Cancer of unknown primary site (CUP) is a heterogeneous group of cancers whose tissue of origin remains unknown after detailed investigation by conventional clinical methods. The number of CUP accounts for roughly 3%–5% of all human malignancies. CUP patients are usually treated with broad-spectrum chemotherapy, which often leads to a poor prognosis. Recent studies suggest that the treatment targeting the primary lesion of CUP will significantly improve the prognosis of the patient. Therefore, it is urgent to develop an efficient method to accurately detect tissue of origin of CUP in clinical cancer research. In this work, we developed a novel framework that uses Extreme Gradient Boosting (XGBoost) to trace the primary site of CUP based on microarray-based gene expression data. First, we downloaded the microarray-based gene expression profiles of 59,385 genes for 57,08 samples from The Cancer Genome Atlas (TCGA) and 6,364 genes for 3,101 samples from the Gene Expression Omnibus (GEO). Both data were divided into training and independent testing data with a ratio of 4:1. Then, we obtained in the training data 200 and 290 genes from TCGA and the GEO datasets, respectively, to train XGBoost models for the identification of the primary site of CUP. The overall 5-fold cross-validation accuracies of our methods were 96.9% and 95.3% on TCGA and GEO training datasets, respectively. Meanwhile, the *macro-precision* for the independent dataset reached 96.75% and 98.8% on, respectively, TCGA and GEO. Experimental results demonstrated that the XGBoost framework not only can reduce the cost of clinical cancer traceability but also has high efficiency, which might be useful in clinical usage.

## Introduction

Cancer of unknown primary site (CUP) is a rare type of tumor whose primary lesion cannot be determined even after a detailed investigation by conventional clinical medical methods (1). CUP only accounts for 3%–5% of all human malignancies and has an annual incidence of approximately 7–12 per 100,000 persons (2, 3). However, it is the fourth leading cause of cancer death, because targeted therapy usually requires knowledge of the tissue origin of cancer (4, 5). It is clear that there is an urgent need for an effective and efficient method of tracing the primary site of CUP patients (6).

Recently, next-generation sequencing technologies have facilitated the usage of biomarker-based personalized CUP therapies (7). With the increasing availability to acquire high-throughput genomic and transcriptomic data, various types of molecular biomarkers have been identified and used in identifying the tissue of origin of CUP (8–12). First, patterns of DNA somatic mutations of a CUP patient in conjunction with the Random Forest algorithm were used to predict cancer tissue of origin (8, 13). However, the prediction accuracy is still not satisfactory, especially for clinical usage. Second, copy number alteration was also used to predict tumor tissue of origin with a deep learning framework (14). Though the accuracy improved, it is not easy to call copy number alteration easily for an individual patient. Third, tissue-specific miRNA and DNA methylation markers combined with the random forest algorithm also achieved good prediction results (11, 15–17). However, DNA methylation pattern is also expensive to achieve, which may restrict its clinical usage. Fourth, mRNA is probably the most studied molecule in detecting tissue of origin of CUP patients, which is usually used together with classification algorithms like naive Bayesian and Tree Boosting (10, 18–20). Fifth, there are also methods combining two or several types of molecular biomarkers to predict tissue of origin of CUP (9, 11). Finally, immunohistochemical and diagnostic methods combined with machine learning or deep learning algorithms were also widely used to detect the primary site of CUP (16, 18, 21, 22).

Microarray data analysis of gene expression files is a high-throughput sequencing approach using sequencing technology (23–25). Since RNA-seq has many advantages over microarray such ability to detect novel transcripts, microarray-based detection of tissue of origin of CUP is more or less ignored by previous studies. However, the advantages of RNA-seq seem not to affect the detection of tissue of origin. In addition, there are plenty of microarray data, and microarray seems to be more robust than RNA-seq. Thus, it still might be worthy to test the ability of microarray data in tracing the primary site of CUP. In this study, we developed microarray-based Extreme Gradient Boosting (XGBoost) models from The Cancer Genome Atlas (TCGA) and the Gene Expression Omnibus (GEO) to infer tissue of origin of CUP. To illustrate the validity and rationality of the model, we further revealed the gene expression level in each cancer type and analyzed the enrichment of the genes used in the models.

## Materials and Methods

**Figure 1:**
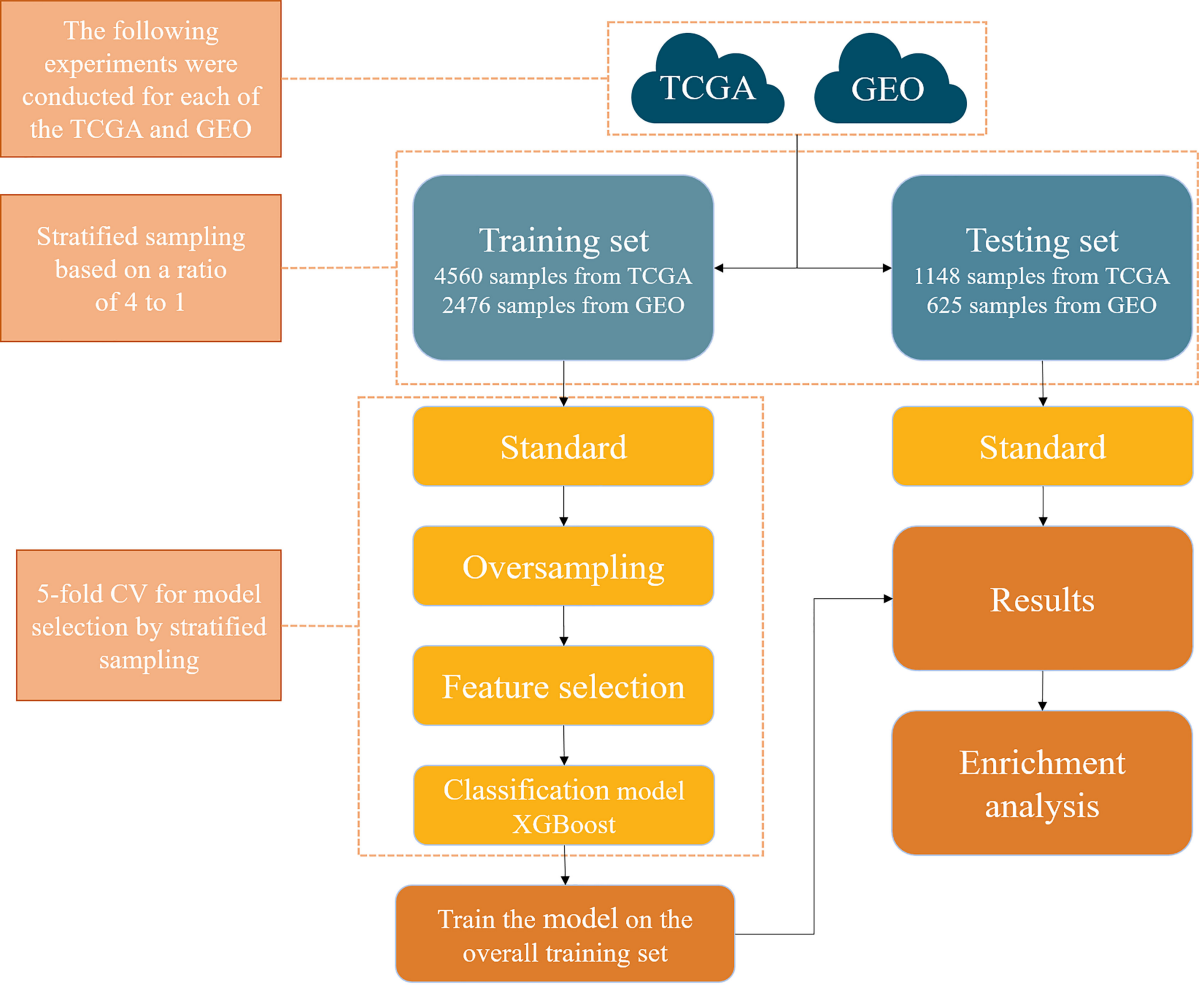
A computational framework to detect the primary site of cancer with an unknown primary lesion.

The framework of this study is shown in **Figure 1**, which consists of a few steps including data collection, feature selection, model construction, and model validation.

### Data Preparation

**Figure 2:**
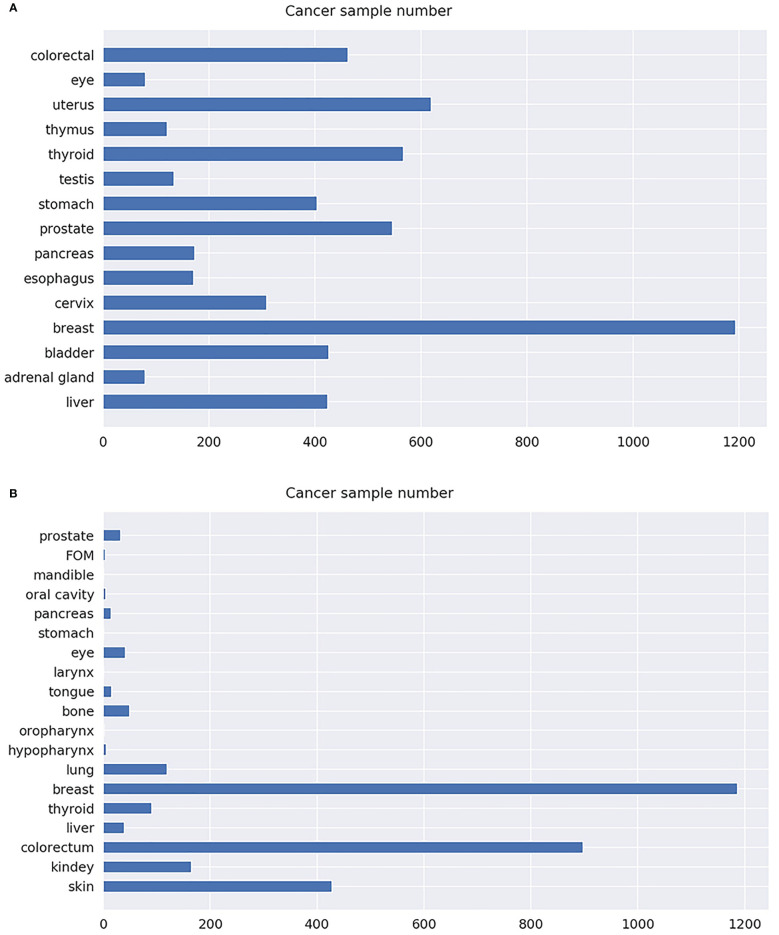
The number of samples for each cancer type. **(A)** T dataset. **(B)** G dataset.

Microarray gene expression datasets of different cancer types were downloaded from TCGA and GEO. The detailed information of the datasets is summarized in **Table 1**. Specifically, a total of 5,708 samples were downloaded from TCGA, covering 15 types of cancer; microarray expression of 59,385 genes for each sample was also retrieved. Similarly, 3,101 samples were also downloaded from GEO, covering 19 types of cancer; microarray expression of 6,364 genes for each sample was also retrieved. The dataset downloaded from TCGA was denoted as T dataset, while that from GEO was denoted as G dataset. Cancers in the hypopharynx, oropharynx, tongue, larynx, stomach, pancreas, oral cavity, mandible, floor of mouth (FOM), and prostate were combined with the other cancer types in the G dataset, due to their small sample size. Therefore, the number of cancer types in dataset G was reduced from 19 to 10. The specific number of samples per cancer of the T and G datasets is shown in **Figure 2**. Detailed information for each sample in the G and T datasets can be found in **Supplementary Table S1**, available online at https://github.com/liqianyue/zeitgeist/tree/main/CUP/dataset.

**Table 1:** Detailed information of the data used in this study.

Data source	Data platform	Number
TCGA microarray data	G450A	5,708
GEO microarray data	GPL570	1,559
	GPL96	629
	GPL10558	304
	GPL10379	269
	GPL1390	185
	GPL5049	155

TCGA, The Cancer Genome Atlas; GEO, Gene Expression Omnibus.

#### Training and Testing Datasets

The training and testing datasets were constructed as follows:

(1) T dataset: Since the numbers of samples in each cancer vary widely, stratified random sampling was used to split the data into the training set and testing set (26). For breast cancer, the samples were randomly distributed to training and testing sets with a ratio of 1:1. For all the other cancers, the ratio was set to 4:1. The reason is that there are ~1,200 samples in breast cancer, and it will dominate the training dataset if the ratio is 4:1. Our general rule is that the ratio is 1:1 if the sample number of a cancer type exceeds 20% total sample size; the ratio is 4:1, otherwise. Finally, 4,202 samples were obtained across 15 cancers in the training set and 1,506 samples in the testing set.
(2) G dataset: By a similar rationale, stratified random sampling was also used to split the data. The ratios for breast cancer and colorectal cancer were set to 1:1, and those for other cancer types were 4:1. Finally, 1,853 samples were obtained across 10 cancers in the training set and 1,248 samples in the testing set.

#### Oversampling

It is worth noting that there is a large difference in the sample number across different cancer types in the training set, which will bias the prediction model. So the Synthetic Minority Oversampling Technique (SMOTE) method was chosen to balance the datasets, which is an oversampling method (27). Specifically, SMOTE first selects a minority class at random and finds its *k* nearest minority class neighbors. The synthetic instance is then created by choosing one of the *k* nearest neighbors *b* at random and connecting *a* and *b* to form a line segment in the feature space.

### Gene Selection Method

**Figure 3:**
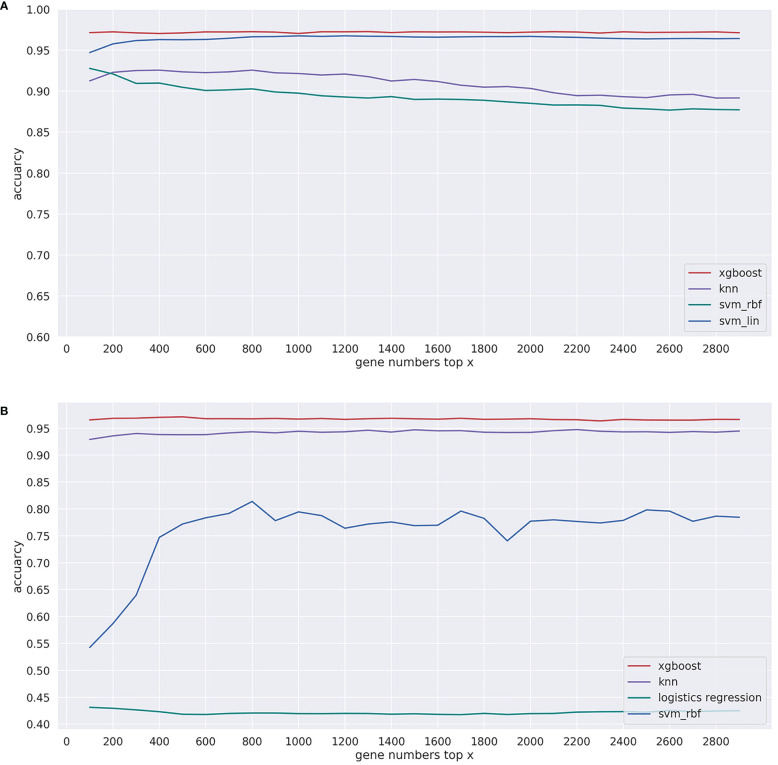
Performance of the model with top x genes in 5-fold cross-validation. **(A)** T dataset and **(B)** G dataset.

For machine learning classification algorithms, irrelevant and redundant features could weaken the effectiveness of the learning algorithm. Therefore, reducing the number of gene features can not only reduce the complexity of the model algorithm and shorten training time but also make the model more generalizable and uneasy to overfit. The gradient boosting algorithm was chosen for gene selection (28). Specifically, the gradient boosting algorithm was first used to calculate the importance score of every gene feature in the T and G training sets. After the importance score was sorted from the largest to smallest, the top *X* (*X* = 10, 20, 30, …, 1000) significant genes were sequentially selected as the input features of models, and then the performance of the model with these *X*-dimensional features in 5-fold cross-validation was recorded (see **Figure 3**). Finally, 200 and 390 genes were determined from each sample in the T and G datasets, respectively, based on the analysis of the 5-fold cross-validation results. The selected gene names can be found in **Supplementary Table S2**, available online at https://github.com/liqianyue/zeitgeist/tree/main/CUP/dataset.

### Model Training

XGBoost is a machine learning system for tree boosting proposed by Chen and Guestrin (29), which has been widely used in the field of bioinformatics in recent years (30–32). For example, Chen and Zhou have used the XGBoost method to trace the primary lesion (19). Similar to this article, XGBoost also has been used to infer the primary lesion of solid tumor types (19) and predict the progression of early-stage prostate cancer in veterans (33). In general, XGBoost is an ensemble model that integrates multiple weak classifiers to reduce the impact of each tree and provide a better learning space.

To obtain the best XGBoost model for solving this problem, three key hyperparameters were selected for fine-tuning during its training within the 5-fold cross-validation based on stratified random sampling. The first parameter “gamma” is the minimum decreasing value of loss function required for node splitting; a high gamma value indicates a more conservative algorithm. The second parameter “max_depth” is the deepest depth of all trees; the larger the max_depth is, the more specific and localized samples the model will learn. The third parameter “min_child_weight” determines the minimum leaf node sample weight sum, which is mainly used to avoid overfitting. When this value is large, it will prevent the model from learning specific local samples.

### Performance Assessment

A 5-fold cross-validation based on stratified random sampling was used to evaluate the classification performance. First of all, the samples of each category were randomly divided into five subsets. Next, one of the subsets was selected as the validation set each time without repetition, and the remaining samples were used as the training set. Finally, the prediction results in the test set (five times) were aggregated and used to measure the prediction performance of the model.

For general classification problems, precision (*P*), recall (*R*), accuracy (*ACC*), and *F*1_*score* were usually adopted to assess the performance of the method. They have been widely used as measurement metrics in previous works (18, 34). They are defined in the following equations. Before that, there was a need to calculate *T_p_*, *T_n_*, *F_p_*, and *F_n_*, whose definitions are shown in **Table 2**.

**Table 2:** Parameters for performance assessment.

Real label	Predict label	
	Positive	Negative
Positive	Ture positive (*T_p_*)	False negative (*F_n_*)
Negative	False positive (*F_p_*)	True negative (*T_n_*)

Then,


$$P=\frac{T_{p}}{T_{p}+F_{p}}$$

$$R=\frac{T_{p}}{T_{p}+F_{n}}$$

$$ACC=\frac{T_{p}+T_{n}}{T_{p}+F_{p}+F_{n}+T_{n}}$$

$$F1\_score=\frac{2\times P\times R}{P+R}$$

Additionally, for multiclassification problems with multiple confusion matrices, macro-average and micro-average were used to evaluate the performance of models (31). Macro-average mainly contains *macro_P*, *macro_R*, and *macro_F1*; similarly, micro-average contains *micro_P*, *micro_R*, and *micro_F1*. Their definitions are shown in the following equations.


$$macro\_P=\frac{1}{n}\sum_{i=1}^{n}P_{i}$$

$$macro\_R=\frac{1}{n}\sum_{i=n}^{n}R_{i}$$

$$macro\_F1=\frac{2\times macro\_P\times macro\_R}{macro\_P+macro\_R}$$

$$micro\_P=\frac{\bar{T_{p}}}{\bar{T_{p}}+{\bar{F}}_{p}}$$

$$micro\_R=\frac{\bar{T_{p}}}{\bar{T_{p}}+{\bar{F}}_{n}}$$

$$micro\_F1=\frac{2\times micro\_P\times micro\_R}{micro\_P+micro\_R}$$

where (*P*_1_, *R*_1_), (*P*_2_, *R*_2_), …, (*P_n_*, *R_n_*) are the precision and recall calculated on the confusion matrix of each class separately. The average of *T_p_*, *T_n_*, *F_p_*, and *F_n_* are obtained by averaging the individual elements of the confusion matrix for all classes, they are recorded respectively as $\bar{T_{P}},\bar{T_{n}},\bar{F_{p}},\bar{F_{n}}$ .

To better measure the classification results of all cancer types, the receiver operating characteristic (ROC) was also drawn, which used the true-positive rate (*TPR*) and false-positive rate (*FPR*) as the horizontal axis and the vertical axis, respectively. In addition, we were interested in the area under the ROC curve, denoted by AUC, which is another commonly used evaluation criterion. The *TPR* and *FPR* are defined in the following equations.


$$TPR=\frac{T_{p}}{T_{p}+F_{N}}$$

$$FPR=\frac{F_{p}}{T_{n}+F_{p}}$$

## Results

### Genes Selected in T Dataset and G Dataset

In the T training dataset and G training dataset, in order to determine the number of genes, we used 5-fold cross-validation based on stratified random sampling to evaluate the performance of the model in the gene selection approach. In this part, we only calculated the overall accuracy; the specific results are shown in **Table 3**. Under each of the two datasets, we bolded the accuracy corresponding to the best performing dimension.

**Table 3:** The influence of gene number to the performance of the XGBoost model based on 5-fold cross-validation.

**Gene number**	**150**	**160**	**170**	**180**	**190**	**200**	**210**	**220**	**230**	**240**	**250**	**260**
T dataset	0.966	0.966	0.966	0.964	0.966	**0.969**	0.967	0.966	0.966	0.967	0.967	0.967
**Gene number**	**340**	**350**	**360**	**370**	**380**	**390**	**400**	**410**	**420**	**430**	**440**	**450**
G dataset	0.947	0.943	0.950	0.950	0.951	**0.953**	0.948	0.849	0.950	0.948	0.950	0.949

Specifically, in the T training dataset, the prediction accuracy of the model was 0.968, when we selected the top 200 genes to train the model. Similarly, in the G dataset, we chose 390 genes, and the accuracy of the model was 0.953. To test the effect of model selections, we compared in **Figure 3** the prediction performances of commonly used machine learning algorithms, including XGBoost, support vector machine with linear kernel function (svm_lin), support vector machine with radial base kernel function (svm_rbf) (35), k-nearest neighbor (knn) (36), and logistic regression (lg) (37).

**Figure 4:**
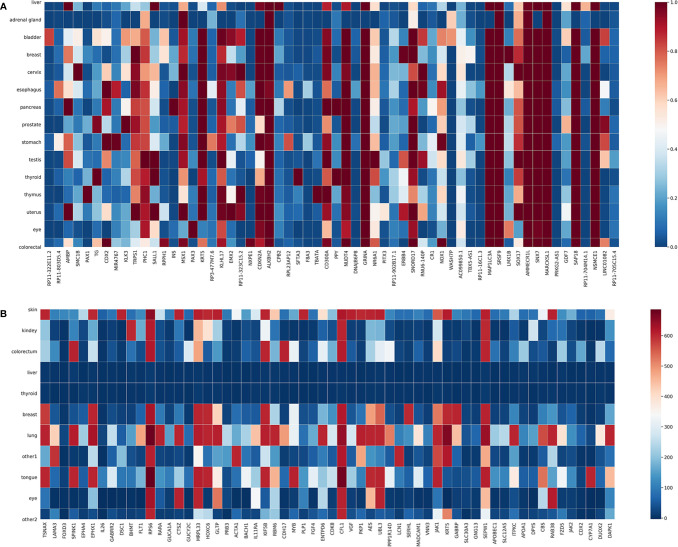
Expression of selected genes in individual cancer types. **(A)** T dataset. **(B)** G dataset.

We also plotted in **Figure 4** the expression levels of the top 60 genes selected from the T and G training datasets in each cancer. Each column in **Figure 4** represents a selected gene; each row represents a cancer type; the color of a block indicates the normalized average expression of a gene in a cancer type. As can be seen, there are a few genes only highly expressed in one cancer type, which might be specific for distinguishing that cancer type. For example, *SMC1B* is highly expressed in cervical cancer. *SMC1B* (Structural Maintenance Of Chromosomes 1B) is a protein-coding gene associated with cell cycle, mitosis, and meiosis. Papasavvas et al. found that *SMC1B* is a feature of cancer precursor dysplasia within high-risk HPV infection (38). There are also other genes highly expressed in several cancer types, indicating that the classification process is quite complex.

### The XGBoost Algorithm Showed the Best Generalization Performance on the Test Dataset

Previously, we selected 200 and 390 genes as final feature inputs of our classifiers from the T and G datasets, respectively. Then, in the T training dataset, we used 5-fold cross-validation with overall accuracy as the model evaluation metric. At last, we obtained the optimum parameters for the final XGBoost model with gamma = 0, max_depth = 12, and min_child_weight = 4. Similarly, in the G training dataset, we obtained the optimum parameters, gamma = 0, max_depth = 19, and min_child_weight = 4.

**Figure 5:**
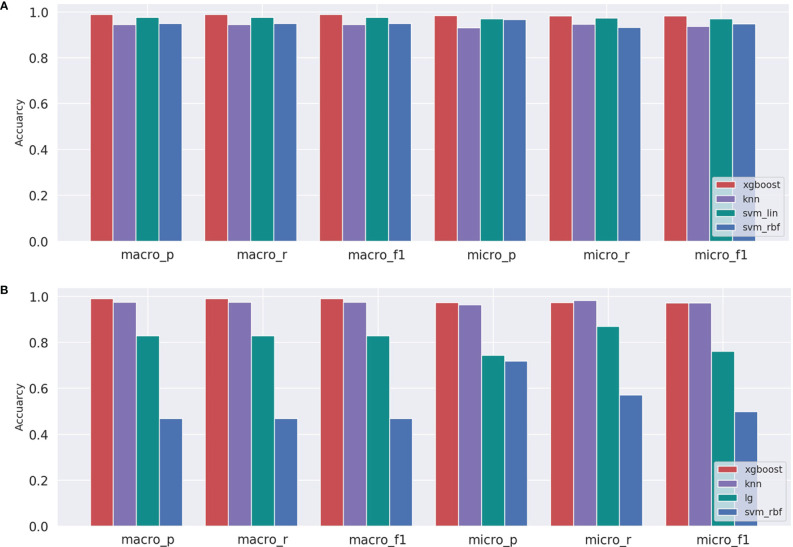
Comparison of machine learning models for independent testing on the **(A)** T and **(B)** G datasets.

**Figure 6:**
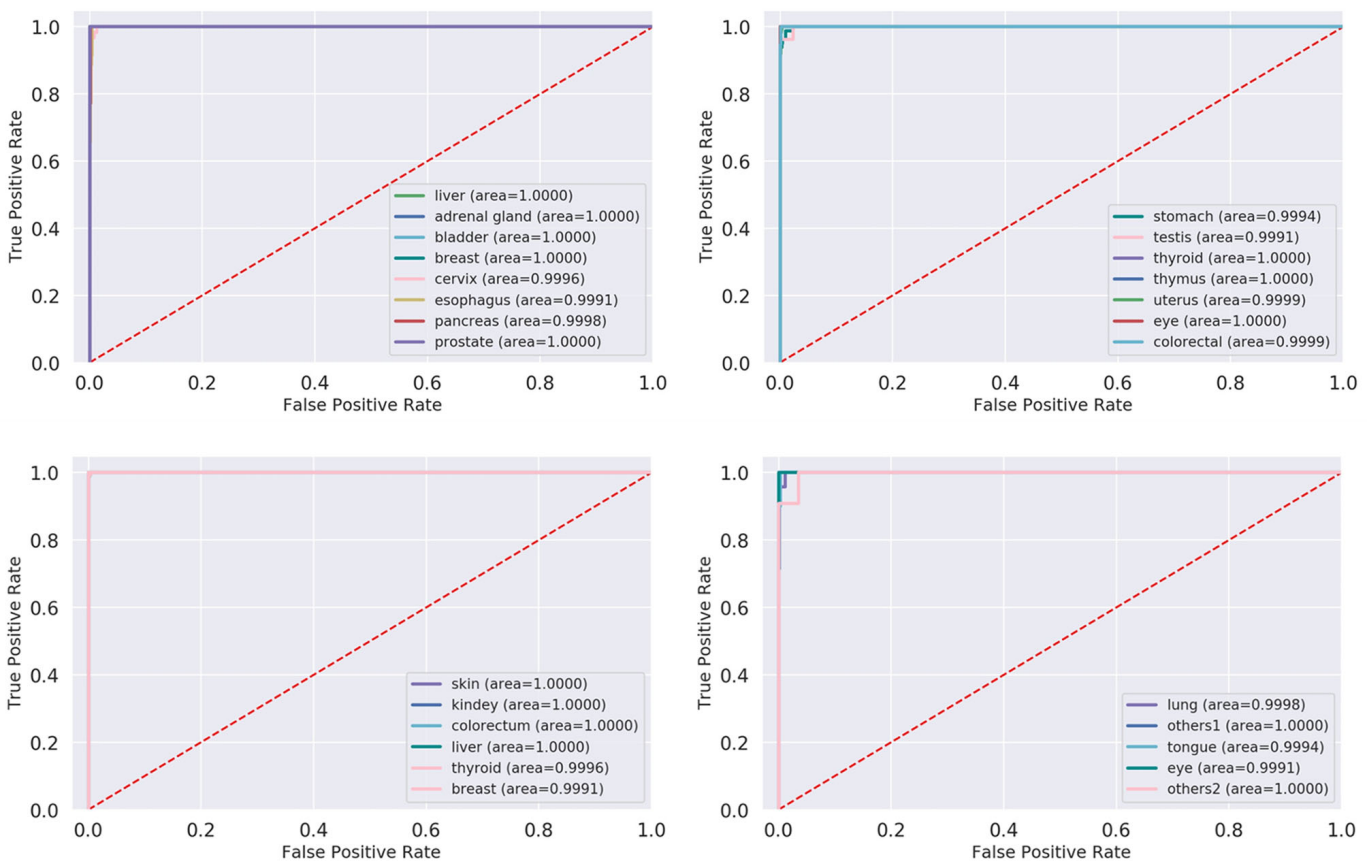
ROC and AUC of XGBoost model in each cancer on test datasets. ROC, receiver operating characteristic; AUC, area under the receiver operating characteristic curve; XGBoost, Extreme Gradient Boosting.

After inputting the best parameters obtained previously into the XGBoost model, we used the full training set data to train the model. Then we used the overall accuracy, macro-average, and micro-average to evaluate the performance of prediction models in the independent test dataset. Furthermore, for the prediction models, we also compared the performances of a few commonly used machine learning algorithms including XGBoost, svm_lin, knn, svm_rbf, and lg. The results of the method comparison are shown in **Figure 5**; the XGBoost model shows the best classification prediction on both the T and G independent test datasets. Furthermore, **Table 4** shows the specific performances of the XGBoost model. Finally, the results of ROC and AUC of the XGBoost model in every type of cancer on independent test datasets are shown in **Figure 6**. As can be seen, XGBoost shows good classification performances on each cancer type.

**Table 4:** The performance of XGBoost model in testing data.

	Macro-P	Macro-R	Macro-f1	Micro-P	Micro-R	Micro-f1
TCGA	0.9675	0.9675	0.9675	0.9531	0.9585	0.9489
GEO	0.9880	0.880	0.9980	0.9530	0.9766	0.9638

TCGA, The Cancer Genome Atlas; GEO, Gene Expression Omnibus.

### Enrichment Analysis

**Figure 7:**
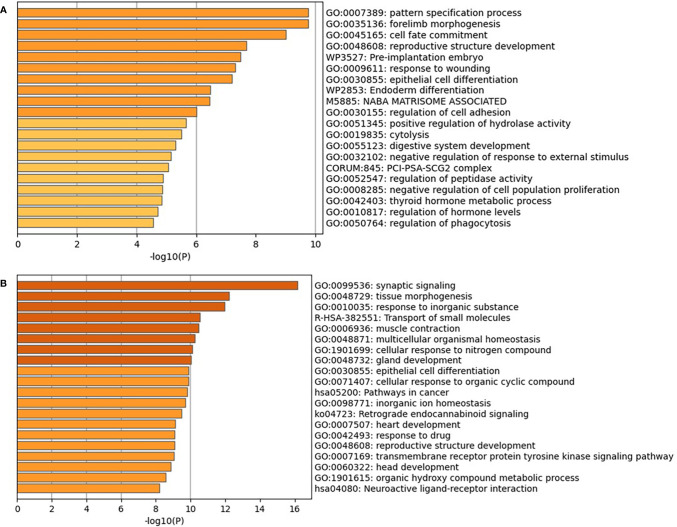
GO and KEGG enrichment analyses of the 200 genes on the T dataset **(A)** and 390 genes on the G dataset **(B)**. GO, Gene Ontology; KEGG, Kyoto Encyclopedia of Genes and Genomes.

**Figure 8:**
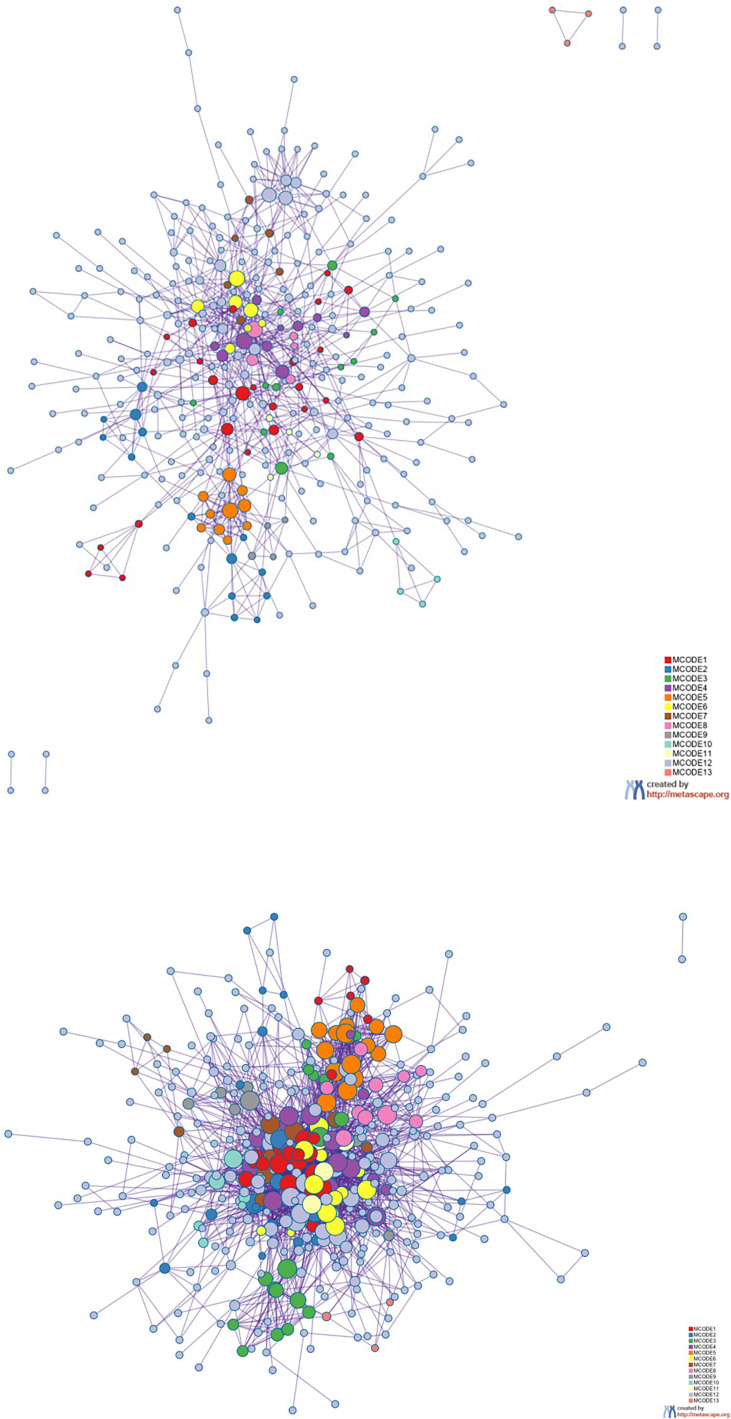
Protein–protein interaction network. The MCODE algorithm was then applied to this network to identify neighborhoods where proteins are densely connected. Each MCODE network is assigned a unique color. The GO enrichment analysis was applied to each MCODE network to assign “meaning” to the network component. GO, Gene Ontology.

For understanding why the selected genes can trace the origin of CUP, we further performed function enrichment analyses on the 200 and 390 genes selected from the T and G datasets, respectively. The results of Gene Ontology (GO) and Kyoto Encyclopedia of Genes and Genomes (KEGG) are shown in **Figures 7** and **8** (39). The enrichment results showed that the genes were significantly enriched in the maintenance and regulation of cell differentiation during morphogenesis of human organs and sub-organ tissues, such as tissue of morphogenesis, regulation of body fluid level, and regulation of system process. Furthermore, the selected genes are highly associated with the development and metastasis of cancer. For example, the aberrant activation of tissue of morphogenesis can also drive distinct stages of cancer progression, including tumor invasiveness, cell dissemination, and metastatic colonization and outgrowth (40). The relationship between hemostasis and malignancy is well recognized, with both elements interacting in a “vicious cycle,” where cancers overexpress procoagulants and thrombin, which in turn promote both prothrombotic potential and tumor growth, invade, and spread (41).

## Discussions

To date, various classification models based on machine learning have been built to trace tissue of origin of CUP. For instance, Chen achieved an average *R* ^2^-score of 96.38% based on XGBoost classification in the RNA-seq datasets of TCGA and the GEO. Liang used the 10-fold cross-validation to evaluate the overall accuracy of naive Bayesian algorithms, which reached 91%. Currently, the prediction for CUP was between 80% and 95%. In 2019, Albaradei with colleagues also proposed a deep learning model called Deep2Met to predict metastatic colorectal cancer using DNA methylation data, which achieved AUC and average F-scores of 0.97 and 0.95, respectively (42).

Although we have made some progress in these studies, there are still some limitations. To be specific, due to the difference in probes between TCGA and the GEO datasets, the two datasets can neither be validated against each other nor be aggregated for use in model training. Moreover, the total number of samples collected for many cancer types was low, resulting in poor model predictions for these types. For example, the accuracies were low for the eyes and adrenal gland on the T dataset, as well as the bone and liver on the G dataset.

Despite that some similar studies have achieved good results, there is still room for improvement. For instance, further research should consider integrating multiple types of biomarkers to improve inference accuracies, such as circulating tumor DNA (43) and H&E images (44). It is also favorable to adopt more advanced machine learning algorithms for prediction or to use algorithms that integrate learning more efficiently (45). In a recent breakthrough, Liu et al. systematically compared the performances of three types of biomarkers including DNA methylation, gene expression profile, and somatic mutation as well as their combinations in inferring the tissue of origin of CUP patients (11). In addition, single-cell RNA sequencing is able to measure the gene expression at the single-cell level, which might further contribute to the accuracy of CUP tissue-of-origin inference (46). Finally, in our current model, there are still other limitations in terms of the source of the cancer data; therefore, it is also a very worthwhile research direction to transfer the model trained on TCGA dataset to the GEO or other datasets.

## Conclusion

In this study, we proposed a machine learning-based approach to detect the primary site of CUP. First, in order to improve the efficiency and prevent over-fitting of models, we selected 200 and 390 genes from all genes on the T and G datasets, respectively. Additionally, we also took heat maps, which is a kind of visualization method, to show the expression level of selected genes. Second, we explored the machine learning frame based on the XGBoost model because the performance evaluation showed that it achieved relatively good results for each cancer type in all models. Finally, we used GO and KEGG enrichment analyses to validate the reasonableness of the gene selection results. In summary, the proposed approach not only can reduce the cost of clinical cancer traceability but also has high efficiency; thus, it is promising in clinical cancer research practice.

## Data Availability Statement

The original contributions presented in the study are included in the https://github.com/liqianyue/zeitgeist/tree/main/CUP. Further inquiries can be directed to the corresponding author.

## Author Contributions

XZ contributed to the conception and design of the study. QFL, FC, and QYL organized the database. QFL, FC, and QYL performed the statistical analysis. QFL, FC, QYL, LC, LT, and GT wrote the first draft of the manuscript. QFL and FC revised the manuscript. All authors contributed to manuscript revision, read, and approved the submitted version.

## Funding

This study was funded by the Natural Science Foundation of Inner Mongolia Autonomous Region of China (Grant No. 2019MS08187).

## Conflict of Interest

The authors GT and QYL are employed by Genesis (Beijing) Co. Ltd.

The remaining authors declare that the research was conducted in the absence of any commercial or financial relationships that could be construed as a potential conflict of interest.

## Publisher’s Note

All claims expressed in this article are solely those of the authors and do not necessarily represent those of their affiliated organizations, or those of the publisher, the editors and the reviewers. Any product that may be evaluated in this article, or claim that may be made by its manufacturer, is not guaranteed or endorsed by the publisher.
